# What works in conservation? Using expert assessment of summarised evidence to identify practices that enhance natural pest control in agriculture

**DOI:** 10.1007/s10531-016-1133-7

**Published:** 2016-05-30

**Authors:** Lynn V. Dicks, Hugh L. Wright, Joscelyne E. Ashpole, James Hutchison, Caitlin G. McCormack, Barbara Livoreil, Klaus Peter Zulka, William J. Sutherland

**Affiliations:** 1 Department of Zoology, University of Cambridge, Cambridge, CB2 3QZ UK; 2 Joint Nature Conservation Committee, City Road, Peterborough, PE1 1JY UK; 3 BirdLife International, The David Attenborough Building, Pembroke Street, Cambridge, CB2 3QZ UK; 4 Swedish University of Agricultural Sciences (SLU), Almas Allé 8, 75007 Uppsala, Sweden; 5 Fondation pour la Recherche sur la Biodiversité (FRB), 195 rue Saint Jacques, 75005 Paris, France; 6 Centre for Evidence-Based Conservation (CEBC), Bangor University, Bangor, Gwynedd LL57 2UW UK; 7 Environment Agency Austria, Spittelauer Lände 5, 1090 Vienna, Austria; 8 Department of Integrative Zoology, University of Vienna, Althanstr. 14, 1090 Vienna, Austria

**Keywords:** Pest regulation, Ecosystem services, Natural enemy, Pest management, Agriculture, Evidence synthesis

## Abstract

This paper documents an exercise to synthesize and assess the best available scientific knowledge on the effectiveness of different farm practices at enhancing natural pest regulation in agriculture. It demonstrates a novel combination of three approaches to evidence synthesis—systematic literature search, collated synopsis and evidence assessment using an expert panel. These approaches follow a logical sequence moving from a large volume of disparate evidence to a simple, easily understandable answer for use in policy or practice. The example of natural pest regulation in agriculture was selected as a case study within two independent science-policy interface projects, one European and one British. A third funder, a private business, supported the final stage to translate the synthesized findings into a useful, simplified output for agronomists. As a whole, the case study showcases how a network of scientific knowledge holders and knowledge users can work together to improve the use of science in policy and practice. The process identified five practices with good evidence of a benefit to natural pest regulation, with the most beneficial being ‘Combine trap and repellent crops in a push–pull system’. It highlights knowledge gaps, or potential research priorities, by showing practices considered important by stakeholders for which there is not enough evidence to make an assessment of effects on natural pest regulation, including ‘Alter the timing of pesticide application.’ Finally, the process identifies several important practices where the volume of evidence of effects on natural pest regulation was too large (>300 experimental studies) to be summarised with the resources available, and for which focused systematic reviews may be the best approach. These very well studied practices include ‘Reduce tillage’ and ‘Plant more than one crop per field’.

**Electronic supplementary material:**

The online version of this article (doi:10.1007/s10531-016-1133-7) contains supplementary material, which is available to authorized users.

## Introduction

**Fig. 1:**
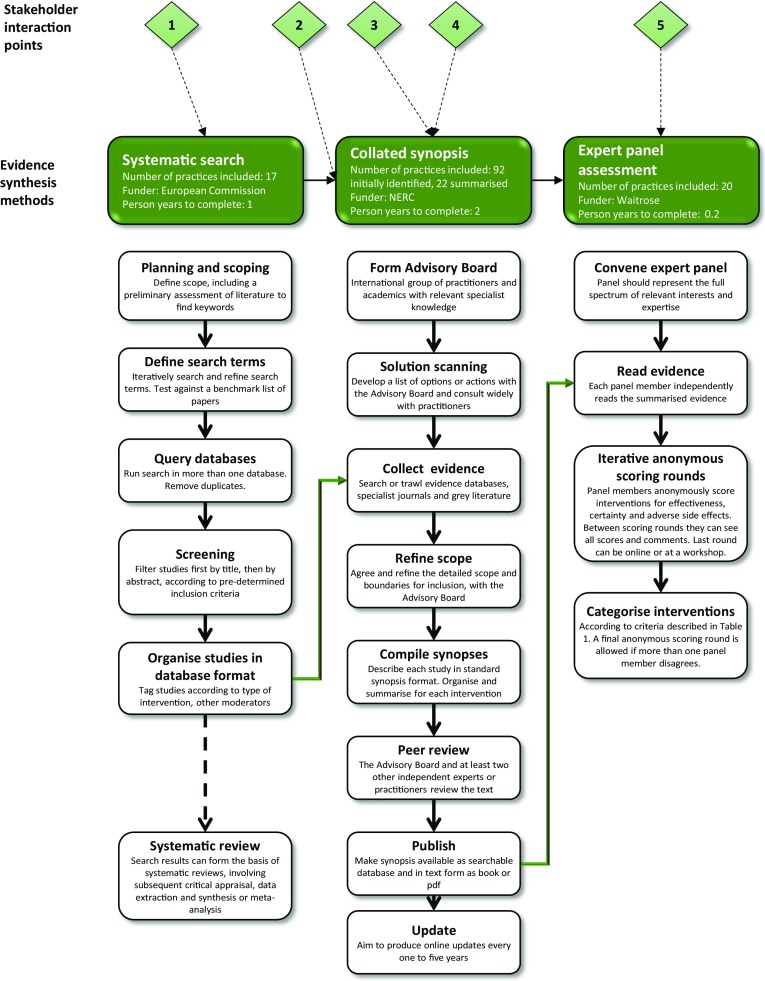
The sequence of methodological stages, showing the funder, the number of practices included and the number of person-years of staff time needed to complete each stage. Stakeholder interaction points are described further in the text. *1*, *2* Selection of topic, *3* input on list of practices from synopsis Advisory Board, *4* prioritisation of practices for summary in the synopsis, *5* expert assessment panel. Beneath each evidence synthesis method is a vertical flow chart showing the process. Green arrows indicate interactions between the methods. The *dashed arrow* indicates that a systematic search can form the basis of a systematic review, a method described by Dicks et al. (2014b) and Pullin et al. (2016), but not used for this case study

This paper describes an exercise to synthesize and assess the best available scientific knowledge on the effectiveness of different farm practices at enhancing natural pest regulation in agriculture. It demonstrates a novel combination of three different approaches to evidence synthesis—systematic literature search (Collaboration for Environmental Evidence 2013), collated synopsis (e.g. Williams et al. 2013) and evidence assessment by expert panel (e.g. Dicks et al. 2014a). Taken together, these approaches follow a logical sequence from a large volume of disparate evidence to a simple, easily understandable answer for use in policy or practice. They fall within the existing framework of the ‘4S’ hierarchy for organising evidence described by Dicks et al. (2014b). The example of natural pest regulation in agriculture was a selected case study within two entirely independent science-policy interface projects between 2012 and 2014. One of these was the European BiodiversityKnowledge project, which is the subject of many papers in this special issue (Nesshöver et al. 2016). The other was a UK-focused Knowledge Exchange Programme on Sustainable Food Production, funded by the Natural Environment Research Council. Here, we document how these projects and a funder from the business community (Waitrose plc) combined resources to produce an output of use to agronomists and policy-makers. The stages in the process, along with the projects that funded them, are shown in Fig. 1. Stakeholders from across industry (including food retailers and farmers), Non-Governmental Organisations (NGOs), policy and academia were involved in shaping the process at five specific interaction points from beginning to end (Fig. 1 and Methods).

### Natural pest regulation as an ecosystem service in agriculture

Natural pest regulation is an important regulating ecosystem service for agricultural production (Maes et al. 2011). It refers to the control, suppression or regulation of unwanted organisms that reduce yield through crop damage, or plant or animal ill-health. As an ecosystem service, this is provided by wild, free-living organisms such as the community of natural enemies—predators, parasites or parasitoids (Letourneau et al. 2009; Griffin et al. 2013). The natural pest regulation service has been valued at $4.5 billion per year for the United States (Losey and Vaughan 2006), at $68–200 ha^−1^ year^−1^ on organic farms, but $0 ha^−1^ year^−1^ in conventional farming systems (Sandhu et al. 2015), or between $1.5 and $12 million in just the cucumber and squash fields of the US states of Georgia and South Carolina (Letourneau et al. 2015).

The effectiveness of methods to enhance natural pest regulation in agriculture is of strong interest to policymakers, farmers and agronomists. These methods represent a key element of ‘ecological intensification’ (Bommarco et al. 2014; Pywell et al. 2015), in which the role of functional biodiversity in delivering production-related ecosystem services such as pollination, soil fertility, water quality and pest regulation is actively managed and enhanced. Enhancing natural pest regulation can enable incremental reductions in the use of synthetic chemicals in crop and livestock protection. Reduced overall use of pesticides in agriculture is a very clear policy aim for the French Government, under its ‘Ecophyto 2018’ strategy (MAAF and MEDDE 2015), and a general policy direction under the European Union Sustainable Use of Pesticides Directive (EU Directive 2009/128/EC), which requires Member States to have national pesticides action plans (Barzman and Dachbrodt-Saaydeh 2011). Reducing pesticide use offers direct benefits to farmers through lower input costs.

Enhancing natural pest regulation is a key aspect of ‘Integrated Pest Management’ (IPM; Brewer and Goodell 2012; Pimentel and Peshin 2014), which is strongly promoted in policy. For example, the recent National Pollinator Strategy for England identifies promoting IPM as one of its main strategic actions, with the aim of reducing the impacts of pesticides on wild and managed pollinators, by reducing use of insecticides, and therefore exposure levels (Defra 2014).

There is a very wide literature on methods to enhance natural pest regulation, such as through habitat or landscape management, or various IPM techniques. For example, Tschumi et al. (2015, 2016) recently demonstrated that flower strips can reduce cereal leaf beetle (*Oulema* sp.) damage to wheat, and enhance wheat yield by 10 %, in fields not treated with insecticide. To our knowledge, the full breadth of this evidence has not previously been brought together in a format readily accessible to policymakers and agronomists, or analysed in the context of ecosystem service delivery. Reviews and meta-analyses that have been published usually focus on one specific aspect, such as the influence of habitat management (Landis et al. 2000) or landscape composition (e.g. Bianchi et al. 2006; Veres et al. 2013; Chaplin-Kramer et al. 2011), or options to manage a specific pest organism (e.g. Kearney et al. 2016), or the community of pests in a specific crop or livestock animal (e.g. Green et al. 2015 on coffee). These reviews can be immensely useful, but they are widely scattered in the scientific literature and can be inaccessible to decision-makers due to publication charges or their complex technical language and level of detail. It is difficult to find direct comparisons of effectiveness among different types of practice, or different farming systems, a common problem when interpreting scientific evidence for decision-making (Smith et al. 2014). There is a need for a synthesis of evidence that looks across a wide range of practices and compares their ability to enhance natural pest regulation.

## Material and methods

### Context and selection of the case study

Natural pest regulation was selected as a focus by two independent science-policy interfaces, linked together through the European network of knowledge holders established by the BiodiversityKnowledge project (Nesshöver et al. 2016; Livoreil et al. 2016).

The BiodiversityKnowledge project itself identified three cases studies to test the process of responding to knowledge needs in support of policy decisions (marine, conservation corridors and agriculture; see Schindler et al. 2016). For the agricultural case study, discussions with the French and the Austrian ministries of ecology and agriculture in 2011–2012 (Fig. 1, stakeholder interaction 1) defined a joint question of interest for policy-makers, finalised as: “Which types of landscape management are effective at maintaining or increasing natural pest regulation”. Using the network of knowledge, a broad consultation was launched in April 2014 to identify a working group to respond to this request.

Concurrently in the UK, the Natural Environment Research Council’s Knowledge Exchange Programme on Sustainable Food Production, led by the University of Cambridge, selected the pest regulation service in agriculture as one of three focus subjects for summarising existing scientific evidence. The Knowledge Exchange Programme aimed to identify subjects where research funded by the Natural Environment Research Council could be used to enhance the sustainability of UK food production through impacts on practices in the agri-food supply chain. Subjects were selected through a process of online consultation with businesses, policy makers and third sector organisations (Fig. 1, stakeholder interaction 2).

Following exchanges between the project leaders, a partnership was established to combine methods and share tasks.

### Selection of methodological approaches

**Fig. 2:**
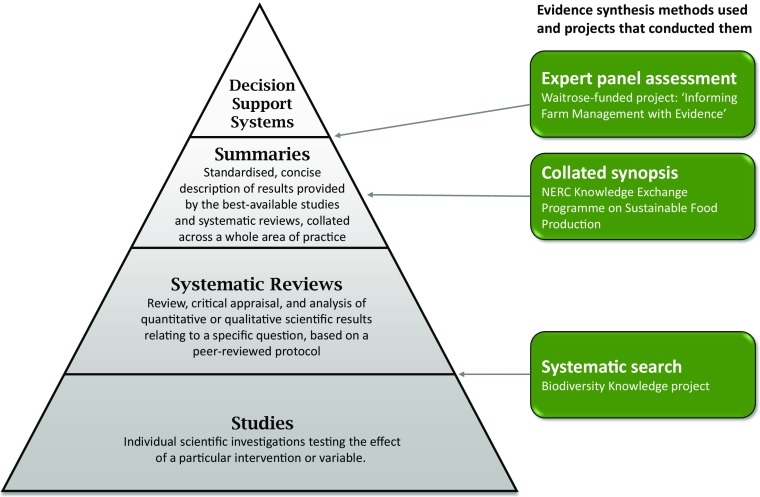
A schematic showing how the evidence synthesis methods used in the case study fit within the ‘4S’ hierarchy for organising evidence for use in environmental decisions. The systematic search method is the first step in systematic reviews; the collated synopsis method is equivalent to the summary level; the expert assessment can be used as part of a summary for decision-makers, but also to synthesize the summary information further for use in decision support systems. Adapted, with permission, from Dicks et al. (2014b)

The policy makers in dialogue with the BiodiversityKnowledge project (knowledge requesters) were eager to get a list of possible practices in natural pest control, as well as a synthesis about evidence of their effectiveness. This led the authors to opt for a systematic map or review approach (Collaboration for Environmental Evidence 2013) among those knowledge synthesis methods described by BiodiversityKnowledge for responding to policy questions (Pullin et al. 2016). This corresponds to the second level in the 4S hierarchy of organising evidence described by Dicks et al. (2014b; shown in Fig. 2).

The NERC Knowledge Exchange Programme on Sustainable Food Production used a ‘collated synopsis’ method developed by the Conservation Evidence project at the University of Cambridge (described by Dicks et al. 2014b) as its approach to summarising evidence. Broad subject areas suitable for this approach were selected by the Programme. Other methods of evidence synthesis, such as systematic review, or use of expert opinion, were not in the scope of the Programme.

The collated synopsis approach corresponds to the summary level in the 4S hierarchy of organising evidence (Fig. 2), because it involves collating brief, plain language descriptions of either studies or systematic reviews across an area of practice, and extracting overall messages or recommendations for decision-makers (see definitions in Dicks et al. 2014b). It is appropriate for a broad subject area that incorporates many different possible actions or research questions. The method of searching the literature for a collated synopsis is flexible (http://www.conservationevidence.com/site/page?view=methods), but must be clearly explained and transparent to maintain rigour and replicability.

As the two projects selected methodological approaches from adjacent levels of the 4S hierarchy (Fig. 2), they articulated together well as a combined work programme. As indicated in Fig. 1, the systematic search became the main source of literature for the collated synopsis.

### Step 1: systematic literature search

Following the guidelines for environmental systematic reviews (Collaboration for Environmental Evidence 2013), a systematic literature search was undertaken by librarians at l’Institut National de la Recherche Agronomique (INRA, France), using CAB Abstract as the main database, complemented by Web of Science (both searched 1973-July 2012). The search equation comprised strings of relevant terms in English, including a comprehensive list of pest groups (from INRA HYPPZ9; http://www7.inra.fr/hyppz/), broad categories of natural enemies, and types of practice and their outcomes (e.g. ‘increase’, ‘decrease’, ‘maintain’ etc.). The search terms were chosen by an iterative process of searching and refining. The final search equation was tested for effectiveness against a benchmark list of 83 papers identified as relevant by a scoping exercise run on Scopus (PZ), Biosis (PZ) and Web of Science (BL), and a preliminary list sent by University of Cambridge (264 titles identified from the content trawl of three journals; see below). The complete list of search terms is provided in Supplementary Information, Part 1. Results of the searches in CAB Abstract and Web of Science were combined and duplicates removed. Titles not mentioning agriculture or ecological topics (e.g. medicine) were discarded (Two authors did this independently; Kappa test for consistency 0.71; Landis and Koch 1977).

The practice terms used in this search focused on maintaining or restoring natural (or semi-natural) habitat, the focus chosen by the BiodiversityKnowledge project. Seventeen categories of practice (e.g. grazing) or habitat features (e.g. shelterbelts) were identified, as indicated by the ‘action’ search terms (Supplementary Information Part 1). This provided literature on a subset of the full practice list generated by the NERC Knowledge Exchange Programme, as practices unrelated to habitat management (such as ‘reduce pesticide use’ or ‘use crop rotation’) were not explicitly considered in the search equations. These actions were captured by the journal trawl approach described below, but received less search effort.

### Step 2: the collated synopsis method

Figure 1 (middle column) describes the process of producing a collated synopsis, as followed by the Conservation Evidence project (http://www.conservationevidence.com). For published examples and more details of the method, see Dicks et al. (2010), Williams et al. (2013), Smith et al. (2014) and Berthinussen et al. (2014). Methodological details of most stages are defined by what is feasible and practical for each specific synopsis, with agreement of the Conservation Evidence project, and are reported in the preamble to each synopsis.

The ‘Solution scanning’ stage (Fig. 1) used a list of practices suggested by ecosystem service experts, presented in Sutherland et al. (2014). This list was refined and added to as the literature was reviewed. An international advisory board of seven experts (from academia, private-sector research and independent and charitable organisations; names listed in Wright et al. 2013, p. 4) also commented on and added to the list (Fig. 1, stakeholder interaction 3). Any practice that farmers or land-managers would realistically be willing or able to carry out was included, regardless of whether it had already been adopted anywhere, or whether or not evidence for its effectiveness existed. The synopsis only included evidence relating to wild natural enemies from within the same natural ecosystem. As a result, it did not include most of the extensive literature on biological control, which usually uses introduced, non-native organisms.

In the ‘Collect evidence’ stage (Fig. 1) papers from the systematic literature search and papers from a full content trawl of three journals—*Agriculture, Ecosystems & Environment*, *Biological Control* and *Journal of Applied Ecology*—were screened based on abstracts. Criteria for the inclusion of a study in the final synopsis were:

1. The study must include a practice that would be done for the purposes of enhancing natural pest regulation or sustainable land management. This excludes studies that look at correlations across different landscapes or habitats, but includes studies comparing sites with historically different active practices.
2. The effects must have been measured quantitatively, and should usually compare with a control treatment or with measurements taken before the practice took place.

Retained studies were tagged according to the practice(s) they tested, and compiled into a Microsoft Access database. This final database could be considered an unpublished systematic map (a catalogue or database of available evidence, as defined in Dicks et al. 2014b; James et al. 2016), and contained 3947 individual studies (see “Results” section). This volume of literature could not be summarised in full with the available resources, so we selected a subset of practices for which to summarise and assess evidence.

To prioritize a subset of practices to be summarised, we asked stakeholders from the food production industry, agricultural policy and academia to select practices for which they would most like to see evidence summarised (Fig. 1, stakeholder interaction 4). A prioritisation exercise was repeated four times with different groups of eight stakeholders (similar methods described in Sutherland et al. 2011), during a workshop in Paris in January 2013. Participants came from several western European countries and were asked to vote privately on their personal top 10 practices and then agree the group’s final top 10 by consensus, after seeing the votes from the first round. The priorities identified were encouragingly consistent between the four groups, with a total of 18 selected across all four groups.^1^ Five priority practices selected by all four groups were included in the collated synopsis, balancing the stakeholder priorities with the project’s time constraints.^2^ Many of the selected priority practices had a large volume of literature (>200 studies), and it would have been impossible to summarise more than one or two of these in full, in the timescale of the funded project (one year in total). Two priority practices with a large literature—‘Use crop rotation’ and ‘Convert to organic farming’—were included in limited form, by summarising a subset of the available evidence (rotations involving potato crops, and experimental but not site comparison organic farm studies). In addition to the five priority practices, seventeen other practices were chosen by the synopsis author team to represent all farming systems and the variety of different types of practice from the complete list. The selected practices were those with relatively small amounts of available evidence, to enable the collated synopsis to be completed with limited available resources.

### Step 3: expert panel assessment process

We conducted an expert assessment of the summarised evidence using a modified version of the Delphi technique (Mukherjee et al. 2015), following a protocol outlined in Sutherland et al. (2015). An expert group completed three rounds of scoring and discussion of the summarised evidence for the shortlisted practices.

The group comprised 16 participants from agri-business, conservation NGOs and academia (Fig. 1, stakeholder interaction 5; see Supplementary Information, Appendix 1 for a list of group members). As recommended for developing ‘Clinical Practice Guidelines’ in evidence-based medicine (Graham 2011), these participants were selected to represent the full range of relevant interests and areas of expertise. They included campaign organisations focused on reducing pesticide use and conserving biodiversity, companies manufacturing agri-chemicals and biological control agents, independent researchers, agronomists and companies involved directly in food production. Eleven of the participants completed the online scoring survey and attended a 1 day workshop at the University of Cambridge in April 2014, to discuss and reconsider their assessments. Four completed the survey, contributed comments for the workshop via email and rescored remotely using an Excel spreadsheet. One participated in the survey stage only.

Each member of the group read the summarised evidence for the selected practices, and independently scored each practice between 0 (low) and 100 (high) for:

1. The effectiveness at enhancing natural pest control.
2. The strength of any potential negative side-effects associated with the practice.
3. The certainty of the evidence about each practice in the synopsis.

Details of the scoring, and guidance given to the assessors, are provided in Supplementary Information, Appendix 3.

During the workshop, participants were presented with the range and median scores of the group. Each practice was discussed in detail and the group members each scored again, anonymously and independently. Important discussion points were recorded and are included in a final guidance document (Supplementary Information, Part 2). Medians of the second round scores were used to place the practices into categories of effectiveness, using thresholds shown in Table 1. In a third and final round of scoring, the experts were asked if they agreed with the categories. If more than one member disagreed, all experts scored again, independently and anonymously and with reference to the summarised evidence, to give the final scores.

**Table 1:** Categories of effectiveness

Thresholds are applied to median percentage scores across an expert panel after at least two rounds of anonymous scoring. Reproduced, with permission, from Sutherland et al. (2015)

## Results

Figure 1 includes information on how many ‘person years’ of staff time was required for each of the evidence synthesis steps described. In total, the process took just over three person years, with the final stage, expert assessment, being the quickest, at 0.2 person years.

### Systematic literature search

The systematic literature search returned 33,852 studies (14,249 from CAB Abstracts and 19,603 from Web of Science) once duplicates were removed. We estimated that these searches obtained approximately 56 % of the relevant literature, based on the percentage of references from the benchmark list that were returned by the searches. This is a relatively low capture percentage, reflecting the difficulty of designing systematic search terms for such a broad question. With more time and resources, it may have triggered further refinement of the search terms, but this was not possible with the resources available. After title screening, a set of 4202 papers where retained to be screened at abstract stage.

## The collated synopsis of evidence

**Fig. 3:**
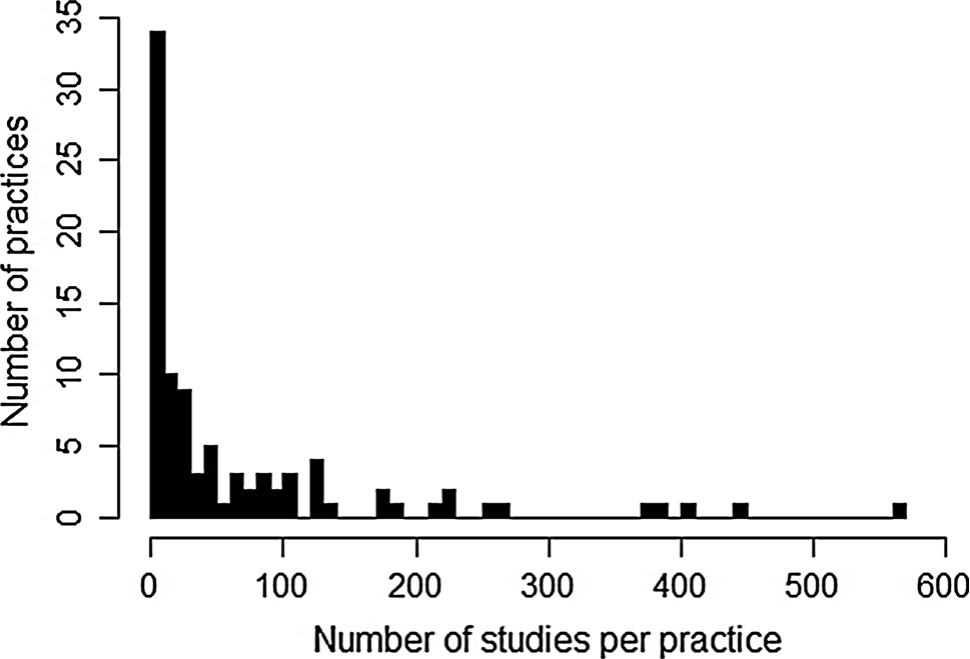
Frequency histogram showing the distribution of number of studies for the 92 practices for which evidence was collected by the systematic search and organised in preparation for the collated synopsis

A set of 92 practices to enhance natural pest regulation in agriculture were identified and 3947 individual studies that tested them were retained. These practices are listed in the Supplementary Information, Appendix 2, which also shows the number of studies testing the effectiveness of each. The number of relevant studies found per practice ranged from 0 to 570, with a strongly positive skewed distribution (Fig. 3). There was just one practice for which no studies were captured by the systematic search—‘Restore or create low-input grassland’—and six practices for which the evidence comprised a single study.

For five practices a very large volume of evidence was collected—over 300 studies. These well-studied practices are: ‘Plant more than one crop per field’ (570 studies), ‘Alter timing of sowing or harvesting’ (445), ‘Reduce pesticide use’ (404), ‘Use crop varieties that resist or suppress pests, diseases or weeds’ (383), and ‘Reduce tillage’ (375). Four of these were selected as priorities during stakeholder interaction 4 (Fig. 1). The exception was ‘Alter timing of sowing or harvesting’. None of these practices was included in the shortlisted subset of practices to be summarised, mainly because the volume of evidence was too large to be summarised with the available resources. There is clearly a need to synthesize evidence for practices such as these, with large numbers of studies and strong interest from stakeholders. The most appropriate method is systematic review, focused on each specific practice (second layer in the 4S hierarchy, Fig. 1). For example, there is currently a systematic review underway for the effects of one of these practices—reduce tillage—on a different ecosystem service, soil carbon (Haddaway et al. 2016). Results of systematic reviews can be summarised in collated synopsis format.

The evidence for the shortlisted subset of 22 practices is fully summarised in Wright et al. (2013), also available as an open access searchable database of practices online (http://www.conservationevidence.com). These practices have between 1 and 19 relevant studies (numbers given in Table 2 for 20 practices assessed at the next stage), with ‘Convert to organic farming’ (19 studies) and ‘Create beetle banks’ (18 studies) having the most.

**Table 2:** Categorisation of a selected subset of 20 practices to enhance natural pest control as an ecosystem service

Category	Interventions falling in this category	Number of studies
Beneficial	Combine trap and repellent crops in a push–pull system	10
Likely to be beneficial	Grow non-crop plants that produce chemicals that attract natural enemies*	4
	Use chemicals to attract natural enemies	15
	Exclude ants that protect pests	7
	Grow plants that compete with damaging weeds	9
Trade-offs	Leave part of the crop or pasture unharvested or uncut	8
	Use crop rotation in potato farming systems	10
Unknown effectiveness	Use pesticides only when pests or crop damage reach threshold levels*	14
	Incorporate parasitism rates when setting thresholds for insecticide use	1
	Alter the timing of insecticide use*	5
	Delay herbicide use	4
	Use alley cropping	8
	Plant new hedges	4
	Allow natural regeneration of ground cover beneath perennial crops	9
	Isolate colonies of beneficial ants	1
	Delay mowing or first grazing date on pasture or grassland	11
Unlikely to be beneficial	Create beetle banks	18
Likely to be ineffective or to have adverse side-effects	Incorporate plant remains into the soil that produce weed-controlling chemicals	10
	Use grazing instead of cutting for pasture or grassland management	8
	Use mixed pasture	7

Based on assessment by an expert panel. Adapted from Sutherland et al. (2015). Practices are placed in the categories using median scores from experts, according to the criteria described in Table 1. Practices marked ‘*’ were selected as priorities for evidence summary during a stakeholder consultation exercise (stakeholder interaction 4, Fig. 1). The final column shows the number of individual studies on which each assessment was based. These studies are cited in Wright et al. (2013), or on the website www.conservationevidence.com, where the collated synopsis on natural pest control is available as a searchable electronic resource

## Evidence assessment for selected practices

During the expert panel assessment, two practices that appear in Wright et al. (2013) were excluded. The practice ‘Convert to organic farming’ was excluded from assessment on the advice of the synopsis Advisory Board, because the synopsis omits a large number of well-known site comparison or correlative studies comparing organic with non-organic farms. This is in spite of it being the practice with the largest number of studies in the collated synopsis. ‘Use mass-emergence devices to increase natural enemy populations’ was excluded by the expert panel during workshop discussions, because it sounds as though it includes the widespread practice of introducing external (sometimes non-native) natural enemies to the system, known as biological control. The extensive literature on biological control was outside the scope of the synopsis unless the organisms used were native. As a result of this very restrictive scope, the practice had only one relevant study in the synopsis, about the control of horse chestnut leaf miners (Kehrli et al. 2005). An evidence assessment of this action on the basis of this single study would have been extremely misleading.

Table 2 shows the list of 20 assessed practices, sorted by category of effectiveness and certainty. An ‘Agronomist’s Guide to Evidence for Selected Practices’ was written, containing the assessment categories, final scores and important discussion points from the meeting. This was provided to the panel and the funder, and is included here as Supplementary Information Part 2.

Only one practice was identified in the most effective ‘beneficial’ category, characterised by high certainty and high effectiveness scores without adverse effects. This practice, the ‘push–pull system’, has been carefully studied in maize and bean crops, in Africa. Well-designed, replicated trials have demonstrated positive effects on natural enemies, reduced pest number, pest damage and increased yields in response to this practice (for example, Khan et al. 2010; see http://www.conservationevidence.com/actions/753, or Wright et al. (2013) for links to all 10 relevant studies). It clearly has strong potential to enhance natural pest regulation.

Of the four practices assessed that were identified as priorities in stakeholder interaction 4 (marked with ‘*’ in Table 2), one falls in the unknown effectiveness category: ‘Alter the timing of insecticide use’. Five relevant studies were captured that tested the effect of this practice on natural pest regulation (see http://www.conservationevidence.com/actions/723 or Wright et al., 2013). As described in the Agronomists’ Guide (Supplementary Information Part 2), there is some evidence that this practice can lead to enhanced natural enemy abundance and a subsequent reduction in pest numbers. The studies are well-designed and there is relatively good global coverage. However, the number of studies is quite small and many did not measure natural enemy numbers, leading to a low certainty score. Overall, the practice shows potential, but effects and appropriate timing are highly context specific. Given the priority given to the practice by stakeholders, there is a very clear need for further research.

## Discussion

This case study began as part of the BiodiversityKnowledge project, and was extended and continued by collaborators from the wider BiodiversityKnowledge network. It showcases a combined methods approach to summarising scientific evidence for practitioners and policymakers. The collated synopsis and assessment stages can be conducted for a subset of practices from a systematic map, as demonstrated here, and the assessment method can be used repeatedly to query the evidence for different questions, or regions. As pointed out by Pullin et al. (2016, this issue), it is useful to benefit from different methods and be able to combine them, to answer a variety of requests.

This approach lends itself well to areas of policy and practice, or science-policy interactions, where there is a need to diagnose threats, select management actions, or decide how to monitor environmental outcomes. These are areas where available scientific information is often disparate, and variable in relevance, quality, and extent. It is less suitable for cases where only very context-specific information is relevant, such as species or ecosystem ecology, status or distributions (Dicks et al. 2014b).

The stakeholders involved at the interaction points described in Fig. 1 and *Methods* were able to exert substantial influence. For example, in step 3 two practices were excluded from the final assessment because stakeholders felt that the best available relevant evidence was not satisfactorily represented (see Results section, *Evidence assessment for selected practices*). In both cases, the decision was well justified. Sets of evidence relevant to practice were obviously missing, due to the constraints and scope of the prior stages of evidence synthesis. Rather than being a limitation of the expert assessment method, stakeholder influence is important, because it generates buy-in to the outcomes from stakeholder groups, and helps to ensure that the outcomes are relevant and understandable. Different stakeholder sets were involved at each of the different stages, creating a broad body of consultees overall and raising awareness of the process among the stakeholder community.

It is important to guard against introduction of bias through stakeholder influence. The processes described do this by using rigorous formal consensus methods such as the modified Delphi technique where possible to avoid undue influence by specific individuals. Changes such as the removal of practices from the assessment had to be clearly and transparently justified, with agreement from all those involved at that stage. For future iterations of a process like this, the set of stakeholders could be more sharply defined, or remain relatively opportunistic to broaden the consultation as widely as possible, depending on the requirements of a particular issue.

This natural pest regulation case study illustrates the value of the Network of Knowledge approach devised by the BiodiversityKnowledge project at linking people and projects together. The pilot synopsis and assessment of evidence were completed using the combined resources of two projects that were initially operating independently, and additional resources from a follow-on project conducted at Cambridge. These efforts were brought together as a result of networking activity.

It is interesting to consider how the stakeholders involved in the final stage of the process—the expert assessment panel—felt about the validity of the exercise and the method of assessment. This group were given an opportunity to provide feedback. Two members of the panel felt the process itself needed more explanation in the output (Supplementary Information), if it is to be used by agronomists. In particular, where the evidence was weak on enhancing natural pest regulation, this was often because the pest regulation service, or impacts of the practices on pest damage or yields had not been measured explicitly. Documented increases in numbers of natural enemies in the wider environment, but not active in crop fields, did not provide a very high level of certainty in this assessment, a subtlety that may not be apparent when using the output.

As an example of this, two members of the panel were uneasy about the categorisation of ‘Beetle banks’ as ‘Unlikely to be beneficial’, arguing that there is still considerable uncertainty about the effect of beetle banks on pest regulation, despite the practice going through the full three rounds of scoring. As explained in the Agronomist’s Guide (Supplementary Information, Part 2), the evidence shows that beetle banks can lead to an increase in natural enemies and a reduction in pests in, or close to, the banks. The six studies that measured natural enemies in the crop found they only penetrated a short distance into the field and only for a limited period of time (Thomas 1991; Thomas et al. 1991; Carmona and Landis 1999; Thomas 2001; Collins et al. 2002; Prasad and Snyder 2006). Enhanced pest regulation within the crop is not strongly demonstrated in the evidence, leading to a low effectiveness score. An assessment of a very similar set of evidence for the effects of beetle banks on farmland biodiversity (as opposed to the pest regulation service) gave a category of ‘likely to be beneficial’, because there is reasonably good evidence that natural enemy numbers are increased within the banks (Sutherland et al. 2015).

Two panel members felt the information would be more useful for the farming and agronomy community if it included details of the specific practices, and used more familiar language, as demonstrated in the following quote from a feedback email:

“Most end users would want to know ‘Does this method mean I get fewer pest problems?’ Or ‘could this method be helpful for reducing my reliance on pesticides or reducing my pest control costs?’ Rather than ‘does this method enhance natural pest regulation?’” Feedback from Expert Panel Member (NGO representative).

There is clearly substantial scope for further work. In the early part of developing the collated synopsis (Step 2), global evidence was mapped for 70 additional practices for which evidence was neither summarised nor assessed (Fig. 3). This includes widely used practices for which there was a large volume of evidence, such as reducing pesticide use (404 studies), or growing more than one crop per field (570 studies), which may be more amenable to systematic review, as discussed above.

The evidence review itself needs updating already, as the searches end in 2012, 4 years ago. New evidence is published continually and it is quite possible that a relatively small number of additional studies published since 2012 would change the overall assessment category for the practices in Table 2, especially for those classed as ‘unknown’ effectiveness’, where there is evidence of benefits (effectiveness score ≥40 %), but not enough to achieve a high certainty score (such as ‘Alter the timing of insecticide use’, median certainty 28 % after two rounds of scoring, discussed in see Results section, *Evidence assessment for selected practices*). In cases where effectiveness score was low but certainty quite high, as in the case of beetle banks discussed above (median effective score 25 %; certainty 60 % after three rounds of scoring), new evidence could shift the category into the beneficial categories, but it would have to carry substantial weight in the judgement of the expert panel members to outweigh the existing body of evidence that led to low effectiveness scores. At least one paper has been published on beetle banks since 2012 (Dekoninck et al., 2013), but as with the 18 studies already assessed, it does not provide evidence of enhanced pest regulation in the crop—only enhanced diversity of carabid beetles within the bank itself. It would therefore be unlikely to change the overall assessment.

The volume of work and staff resources required to compile evidence for such a broad topic might be considered too much to make this a useful approach for all environmental policy. However, if processes are carefully designed to be efficient and cumulative, the cost diminishes and the value increases over time. Updating collated synopses after 5 years is estimated to require 20 % of the cost of the original synopsis (Dicks et al. 2014b), and the expert assessment stage is relatively rapid, once evidence is summarised (Fig. 1). The potential benefits of the investment are greatly increased use of the best available scientific evidence by stakeholders across industry, conservation and policy, enabling better informed and more effective decision making.

## Electronic supplementary material

Below is the link to the electronic supplementary material.

Supplementary material 1 (DOCX 101 kb)

## Footnotes

1 These 18 priority practices are marked * in Supplementary Information, Appendix 2.

2 These 5 practices are those marked *^ in Supplementary Information, Appendix 2.
