# Supplementary material for: What works in conservation? Using expert assessment of summarised evidence to identify practices that enhance natural pest control in agriculture
Source: Biodivers Conserv. 2016 May 30;25(7):1383–99. doi: 10.1007/s10531-016-1133-7 (PMC7175675; doi:10.1007/s10531-016-1133-7)
Supplement: Supplementary file 1 — Supplementary material 1 (DOCX 101 kb) [file 10531_2016_1133_MOESM1_ESM.docx]

**Supplementary Information**

**What works in conservation? Using expert assessment of summarised evidence to identify effective actions - a case study on natural pest control in agriculture**

Lynn V. Dicks^1*^, Hugh L.Wright, ^2^, Joscelyne E. Ashpole, ^3^, James Hutchison ^2^, Caitlin G. McCormack ^4^, Barbara Livoreil ^5,6^, Klaus Peter Zulka,^7,8^ & William J. Sutherland ^1^

^1^ Department of Zoology, University of Cambridge. CB2 3QZ, UK

^2^ Joint Nature Conservation Committee, City Road, Peterborough, PE1 1JY, UK

^3^ BirdLife International, The David Attenborough Building, Pembroke Street, Cambridge, CB2 3QZ

^4^ Swedish University of Agricultural Sciences (SLU), Almas Allé 8, 75007, Uppsala, Sweden

^5^ Fondation pour la Recherche sur la Biodiversité (FRB), 195 rue Saint Jacques, 75005, Paris, France.

^6^ Centre for Evidence-Based Conservation (CEBC), Bangor University, Bangor, Gwynedd LL57 2UW, UK

^7^ Environment Agency Austria, Spittelauer Lände 5, A-1090 Vienna, Austria

^8^ Department of Integrative Zoology, University of Vienna, Althanstr. 14, A-1090 Vienna, Austria

*Corresponding author: lvd22@cam.ac.uk, tel: +00 44 (0)1223 761362

**Contents:**

Part 1 Search equation used for the systematic search

Part 2 Agronomists guide to Evidence for Selected Practices to Enhance Natural Pest Regulation

Appendix 1: Background to study and methodology

Appendix 2: Complete list of 92 natural pest control practices

Appendix 3: Explanation of scoring and detail of experts’ scores for each practice

Appendix 4: Excluded practices

**Part 1: Search equation used for the systematic search**

The search equation for obtaining studies from CAB Abstracts (and secondarily the Web of Science) combined three refined search strings. Studies were selected when at least one term from each of the three strings (descriptors (DE) or topics (TO)) was found in (search equation = String 1 AND String 2 AND String 3).

**Search String 1: Natural Enemies and Pests**

This string combines the list of scientific genus names of pests OR a list of natural enemies as defined by broad categories.

| 1a. | DE=(predatory insects OR predatory arthropods OR predatory birds OR predatory mites OR natural enemies OR predators OR Biological control agent OR pest OR predator prey relationships OR pests) |
| --- | --- |
| **OR** |  |
| 1b. | TO=(*Acalitus* OR Acanthoscelides OR AcidiaOR Aclypea OR Acrolepiopsis OR Aculops OR Aculus OR Acyrthosiphon OR Adoxophyes OR Aegeria OR Aglaope OR Agriotes OR Agromyza OR Agrotis OR Aleurolobus OR Aleurothrixus OR Anarsia OR Anthonomus OR Aonidiella OR Aphanostigma OR Aphelenchoides OR Aphelenchus OR Aphidula OR Aphis OR Apion OR Apodemus OR Arammichnus Archips OR Argyrotaenia OR Arion OR Aspidiotus OR Athalia OR Atomaria OR Aulacaspis OR Aulacorthum OR Autographa OR Bemisia OR Blaniulus OR Blitophaga OR Brachycaudus OR Brachycorynella OR Brevicoryne OR Bruchus OR Byturus OR Cacoecia OR Cacopsylla OR Calepitrimerus OR Capitophorus OR Capnodis OR Capua OR Carduelis OR Cecidophyes OR Cecidophyopsis OR Ceratitis OR Ceroplastes OR Ceuthorhynchus OR Chaetosiphon OR Chromaphis OR Chrysomphalus OR Cirphis OR Clysia OR Cnephasia OR Coenorhinus OR Colaspidema OR Coleophora Colomerus Columba OR Conorhynchus OR Contarinia OR Coroebus OR Corvus OR Corylobium OR Cossus OR Crioceris OR Cryptomyzus OR Curculio OR Cydia OR Dactylosphaera OR Dacus OR Dasineura OR Delia OR Deroceras OR Dialeurodes OR Ditylenchus OR Dysaphis OR Dysaulacorthum OR Empoasca OR Eotetranychus OR Epidiaspis OR Eriophyes OR Eriosoma OR Eulecanium OR Euparypha OR Euphyllura OR Eupoecilia OR Eurydema OR Euxoa OR Euzophera OR Forficula OR Frankliniella OR Fringilla OR Geoktapia OR Globodera OR Gortyna OR Grapholitha OR Gryllotalpa OR Gymnoscelis OR Haltica OR Haplodiplosis OR Haplothrips OR Hapsidolema OR Harpalus OR Hedya OR Helicoverpa OR Heliothis OR Helix OR Heterodera OR Homoeosoma OR Hoplocampa OR Hyalopterus OR Hylemyia OR Hypera OR Hyperomyzus OR Hypoborus OR Hyponomeuta OR Icerya OR Jacobiasca OR Kakothrips OR Korscheltellus OR Laspeyresia OR Lepidosaphes OR Leptinotarsa OR Leptohylemyia OR Lepus OR Leucoptera OR Limothrips OR Liothrips OR Liriomyza OR Lobesia OR Lycophotia OR Lyonetia OR Macrosiphum OR Mamestra OR Melanaphis OR Melanchra OR Meligethes OR Meloidogyne OR Melolontha OR Metatetranychus OR Metopolophium OR Metriochroa OR Micractis OR Microtus OR Monostira OR Mythimna OR Mytilococcus OR Myzocallis OR Myzus OR Nasonovia OR Oberea OR Oecophyllembius OR Operophtera OR Ophiomyia OR Ophonus OR Oryctolagus OR Oscinella OR Ostrinia OR Otiorhynchus OR Oulema OR Palomena OR Palpita OR Pammene OR Pandemis OR Panonychus OR Parahypopta OR Parus OR Passer OR Passerinia OR Pegomyia OR Pemphigus OR Peribatodes OR Phasianus OR Philophylla OR Phloeotribus OR Phorbia OR Phorodon OR Phthorimaea OR Phyllocoptes OR Phyllonorycter OR Phyllotreta OR Phytocoptella OR Phytometra OR Phytonemus OR Phytonomus OR Phytoptus OR Pica OR Pieris OR Platyparea OR Plutella OR Polia OR Polyphylla OR Pratylenchus OR Prays OR Prolasioptera OR Protrama OR Pseudaulacaspis OR Psila OR Psylliodes OR Pyrrhula OR Quadraspidiotus OR Radopholus OR Resseliella OR Rhagoletis OR Rhopalosiphum OR Rhynchites OR Ruguloscolytus OR Saissetia OR Scaphoideus OR Scotia OR Scrobipalpa OR Scutigerella OR Sesamia OR Sitobion OR Sitodiplosis OR Sitona OR Sparganothis OR Spilonota OR Spodoptera OR Stephanitis OR Stigmella OR Sturnus OR Sus OR Synanthedon OR Talpa OR Tetranychus OR Thrips OR Tipula OR Toxoptera OR Trialeurodes OR Trichodorus OR Tylenchulus OR Vasates OR Vespa OR Vesperus OR Vespula OR Viteus OR Xiphinema OR Xyleborus OR Yponomeuta OR Zabrus OR Zeuzera OR Zophodia) |

**Search String 2: Actions**

| 2a. | DE=(Companion crops OR Farming systems OR grassland* OR Border effects OR forest borders OR Intercropping OR crop management OR cropping systems OR crop establishment OR habitats OR territory OR biotopes OR hedges OR Landscape OR land use OR fallow OR strip* OR linear plantations OR shelterbelts OR ground cover OR trap crops OR Tillage OR agricultural land OR interspecific competition OR grazing OR cultural control) |
| --- | --- |
| **OR** |  |
| 2b. | TO=("Banker plant* system*" OR "companion vegetation*" OR "companion plant* " OR "Buffer width*" OR "buffer zone*" OR corridor* OR "field margin*" OR farmscaping OR "integrated production" OR "repellent plant*" OR "spatial arrangement*" OR "set-aside" OR "set aside" OR refuge OR Compost* OR "integrated crop management" OR habitat OR "crop system" OR groundcover OR "flowering borders" OR landscape OR interplanting |

**Search String 3: Outcomes**

| 3. | TO=((increas* OR decreas* OR declin* OR regulat* OR impact* OR variabilit* OR reduc* OR effect* OR intensit* OR sustain* OR maintain* OR support* OR chang* OR enhanc* OR affect* OR abundance) SAME (abundance OR "population size" OR presence OR "species richness" OR "species diversity" OR biocontrol OR "pest control")) |
| --- | --- |

**Part 2: Agronomists’ Guide to Evidence for Selected Practices to Enhance Natural Pest Regulation**

This guide is aimed at agronomists and farm advisors. It presents an assessment of the effectiveness of 22 selected practices for enhancing natural pest regulation on farmland, based on evidence from scientific studies in the [Conservation Evidence Natural Pest Control Synopsis.](http://www.conservationevidence.com/synopsis/download/10http:/www.conservationevidence.com/synopsis/download/10) The assessment was made by a group of 16 pest regulation experts from agribusiness, academia and non-governmental organisations who participated in a number of scoring and discussion stages. The outcomes were used to put the practices into six categories – see the table below – which give an indication of their likely effectiveness. More information on each of the practices and the expert assessment of them is given in the rest of the document. The methodology and a list of group members are in Appendix 1. The practices assessed here are a subset of a longer list of 92 practices in Appendix 2.

| **Categorisation of practices based on effectiveness in enhancing natural pest regulation** | |
| --- | --- |
| **Beneficial** | - [Combine trap and repellent crops in a push-pull system](#Intervention43) |
| **Likely to be beneficial** | - [Grow non-crop plants that produce chemicals that attract natural enemies](#Intervention34) - [Use chemicals to attract natural enemies](#Intervention35) - [Exclude ants that protect pests](#Intervention52) - [Grow plants that compete with damaging weeds](#Intervention63) |
| **Trade-offs** | - [Leave part of the crop or pasture unharvested or uncut](#Intervention33) - [Use crop rotation in potato farming systems](#Intervention42) |
| **Unknown effectiveness** | - [Use pesticides only when pests or crop damage reach threshold levels](#Intervention21) - [Incorporate parasitism rates when setting thresholds for insecticide use](#Intervention22) - [Alter the timing of insecticide use](#Intervention23) - [Delay herbicide use](#Intervention24) - [Use alley cropping](#Intervention31) - [Plant new hedges](#Intervention32) - [Allow natural regeneration of ground cover beneath perennial crops](#Intervention51) - [Isolate colonies of beneficial ants](#Intervention53) - [Delay mowing or first grazing date on pasture or grassland](#Intervention61) |
| **Unlikely to be beneficial** | - [Create beetle banks](#Intervention41) |
| **Likely to be ineffective or to have adverse side-effects** | - [Incorporate plant remains into the soil that produce weed-controlling chemicals](#Intervention44) - [Use grazing instead of cutting for pasture or grassland management](#Intervention62) - [Use mixed pasture](#Intervention64) |

# Expert assessment of each practice

Each practice has a table of median scores from the expert group assessment, which are explained in Appendix 3. A summary of important discussion points from the assessment process is also given. These provide important context for the numerical scores.

**Combine trap and repellent crops in a push-pull system**

**Description:** Push-pull systems involve intercropping the main crop with plants that are repellent to pests (the ‘push’) while also growing plants (trap crops) that are attractive to pests around the main crop (the ‘pull’). This combination of repellent and attractive companion plants keeps invertebrate pests away from the crop and may provide additional benefits through improved habitat and resources for natural enemies. Push-pull systems can also be designed to suppress weeds at the same time as controlling pests. Ground-dwelling invertebrates are frequently surveyed using pitfall traps – small pots buried in the ground up to their rim and left empty or filled with liquid preservatives or water.

| **Practice** | **Assessment by expert group** | | | **Category** |
| --- | --- | --- | --- | --- |
|  | **Effectiveness** | **Certainty of evidence** | **Negative side-effects** |  |
| [Combine trap and repellent crops in a push-pull system](http://www.conservationevidence.com/actions/753) | 70 | 68 | 5 | Beneficial |

**Crops studied** were maize and beans

**Summary of discussion points:**This practice is shown to be highly effective in the crop systems it has been studied in. Results show increased parasitism, reduced pest abundance and reduced weed incidence. Findings are consistent across a good number of well-designed studies (replicated, controlled, and usually randomized, trials). There is some indication that there are some initial set-up costs but also evidence that yield and profit can increase.

***Key things to note:***The evidence – and application of this practice – is limited to maize systems in Kenya and South Africa. There is a need for significant broader research into the applicability of this practice.

**Grow non-crop plants that produce chemicals that attract natural enemies**

**Description:** This action involves growing non-crop plants which produce volatile chemicals (quickly evaporating scents or odours) that attract natural enemies, thereby encouraging the enemies to the main crop. Non-crop plants could be grown in field margins or interspersed into the main crop. Lab studies demonstrating an attractive effect of a plant species or variety to a natural enemy are also included.

| **Practice** | **Effectiveness** | **Certainty of evidence** | **Negative side-effects** | **Category** |
| --- | --- | --- | --- | --- |
| [Grow non-crop plants that produce chemicals that attract natural enemies](http://www.conservationevidence.com/actions/724) | 68 | 40 | 0 | Likely to be beneficial |

**Crops studied** were sorghum, safflower, orange and lettuce.

**Summary of discussion points:**

All of the studies on this practice show an increase in natural enemies or a reduction in pests or pest damage. Some also indicate an increase in yields. There is a reasonable amount of evidence and the studies are well-designed. However, all of the trials use small-scale plots. At a larger scale, it is likely that the effects could be diluted or lost so it is uncertain whether the practice would still be effective when applied at a field-scale. It will be effective in smaller holdings. No side-effects are reported.

***Key things to note:***

The evidence shows that the practice is very effective for small plots. More research is needed into whether it is effective at a larger field scale.

**Use chemicals to attract natural enemies**

**Description:** This involves using chemicals to lure natural enemies into a crop. Communication chemicals of insects and plants (known as pheromones and volatiles, respectively) can be manufactured and deployed to manipulate invertebrates. Examples include the volatiles produced when plants are attacked by pests (e.g. methyl salicylate) and the alarm and sex pheromones of pests or natural enemies, as well as organic extracts from crop or plant leaves. Chemicals are sprayed onto crops or deployed in dispensers placed at regular intervals in the crop. Many studies have tested the efficacy of chemicals by applying them as baits in insect traps such as delta traps (plastic structures hung from branches or posts containing a sheet of sticky paper). Ground-living invertebrates can be sampled by suction sampling, using a vacuum to suck-up and collect specimens for a given time or area of ground.

| **Practice** | **Effectiveness** | **Certainty of evidence** | **Negative side-effects** | **Category** |
| --- | --- | --- | --- | --- |
| [Use chemicals to attract natural enemies](http://www.conservationevidence.com/actions/754) | 40 | 50 | 15 | Likely to be beneficial |

**Crops studied** were apple, banana, bean, broccoli, Chinese cabbage, cotton, cowpea, cranberry, grape, grapefruit, hop, maize, oilseed, orange, tomato, turnip and wheat.

**Summary of discussion points:**

The studies show a consistent positive effect of this practice in enhancing natural enemy numbers and parasitism rates. The trials are all in small-scale plots so there is uncertainty about the effectiveness of the practice at a larger field-scale. There was some suggestion that effects may be concentrated near the chemical lures, so effects may be lessened in larger fields. One study found an increase in pest species, so there is some potential for adverse side-effects. The studies are well designed and show good consistency of results, so certainty of the evidence is relatively high.

***Key things to note:***

This practice shows good potential and its effectiveness has been demonstrated at a small-scale, but it needs to be trialled at a field scale.

**Exclude ants that protect pests**

**Description:** This involves applying adhesive substances or chemicals to the trunks of perennial crop trees, preventing pest-protecting ants from reaching the branches. Many ants form mutualistic relationships with insect pests (e.g. feeding on honeydew secreted by bugs (Hemiptera) such as aphids), defending them from predators and parasitoids. Excluding these ants may therefore increase predation and parasitism rates by beneficial invertebrates. See also 'Isolate colonies of beneficial ants' for managing ants that act as natural predators and improve pest control.

| **Practice** | **Effectiveness** | **Certainty of evidence** | **Negative side-effects** | **Category** |
| --- | --- | --- | --- | --- |
| [Exclude ants that protect pests](http://www.conservationevidence.com/actions/886) | 40 | 50 | 12 | Likely to be beneficial |

**Crops studied** were cherimoyas, cherry, grape, grapefruit, orange, pecan and Satsuma.

**Summary of discussion points:**

This practice shows moderate to good success in enhancing natural enemies and reducing pest species. However, the effectiveness and applicability of the practice may be very narrow as it relies on specific ecological interactions between the ant and pest species. Negative side-effects may result from the adhesive substances used to exclude ants also excluding crawling natural enemies such as earwigs. There is relatively good certainty about the practice due to a large number of well-designed studies (controlled, replicated, randomised) across seven crop types.

***Key things to note:***

The practice shows good potential but more research is needed. It may have very narrow applicability depending on how common these relationships between ants and pest species are and effectiveness may be location and crop specific. Effectiveness can be maximised, and negative side-effects minimized, by using the more effective exclusion methods than the ‘sticky bands’ used in the studies here.

**Grow plants that compete with damaging weeds**

**Description:** This action involves planting species that out-compete damaging weeds, suppressing them by reducing their ground cover, growth or reproduction rate, or by increasing their mortality. The action is generally applied to pastureland or uncropped areas such as field margins and buffer strips. Plants grown to suppress weeds on large parts of arable land are not included here but are relevant to cover cropping practices, e.g. ‘Grow cover crops when the field is empty’, ‘Grow cover crops beneath the main crop (living mulches) or between crop rows’, ‘Grow crops in strips within a cover crop’ and ‘Incorporate leys into crop rotation’ (practices for inclusion in a future synopsis).

| **Practice** | **Effectiveness** | **Certainty of evidence** | **Negative side-effects** | **Category** |
| --- | --- | --- | --- | --- |
| [Grow plants that compete with damaging weeds](http://www.conservationevidence.com/actions/722) | 70 | 60 | 5 | Likely to be beneficial |

**Crops studied** were clovers, fescues, ryegrass, other grasses and turnip.

**Summary of discussion points:**

This practice is consistently shown to suppress weeds. It was found to be effective in all studies in the synopsis. There are several well-designed studies, many long-term, that all show similar positive effects of this practice. However, approximately half of the studies were pot experiments in greenhouses, so there is some uncertainty about how effective this practice would be under field conditions.

***Key things to note:***

Much of the evidence comes from greenhouse experiments. The effectiveness of this practice in the field is less certain and more in-field trials are needed.

**Leave part of the crop or pasture unharvested or uncut**

**Description:** This action involves harvesting or cutting part of a crop field or pasture, often by leaving uncut strips. In pasture, fodder or perennial crops, these strips may be harvested later in a rotation system. The uncut areas provide a refuge for predators from harvesting itself, as well as providing habitat once the rest of the field is cut. This maintains predator populations and enables them to recolonize the following crop.

| **Practice** | **Effectiveness** | **Certainty of evidence** | **Negative side-effects** | **Category** |
| --- | --- | --- | --- | --- |
| [Leave part of the crop or pasture unharvested or uncut](http://www.conservationevidence.com/actions/725) | 45 | 50 | 25 | Trade-offs |

**Crops studied** were alfalfa and meadow pastures.

**Summary of discussion points:**

In all studies in the synopsis except one, there is evidence that this practice successfully enhances natural enemy numbers. However, there is also some suggestion that pest numbers can also increase, so there is a middling score for negative side-effects. The certainty of evidence is relatively high because the evidence based includes a good number of well-designed replicated trials, which show a consistent trend of enhanced natural enemy numbers.

***Key things to note:***

The evidence is limited mainly to Alfalfa. There is good potential for the effectiveness of this practice in other systems, but more research is needed.

**Use crop rotation in potato farming systems**

**Description:** Crop rotation involves alternating between two or more commercial arable crops in successive growing seasons. It may also include ley or fallow periods, as long as at least two crops are involved. Growing different crops each year may help avoid the build-up of crop-specific pests and pathogens.

**NOTE:** The studies presented for this synopsis are only those that test rotations including potato *Solanum tuberosum*. There were approximately 200 further studies on crop rotation in other crop types which will be summarized in the future.

| **Practice** | **Effectiveness** | **Certainty of evidence** | **Negative side-effects** | **Category** |
| --- | --- | --- | --- | --- |
| [Use crop rotation in potato farming systems](http://www.conservationevidence.com/actions/719) | 50 | 50 | 25 | Trade-offs |

**Crops studied** were alfalfa, barley, broccoli, brown mustard, buckwheat, cotton, lupins, maize, oats, pearl millet, peas, potato, rye, sorghum, soybean, sugar beet, timothy grass, wheat and yellow sweet clover.

**Summary of discussion points:**

There is some evidence that this practice can reduce pests and disease in potato crops. The effects vary depending on the rotation being used and the pest species or disease variety. It is particularly effective for controlling Colorado Potato Beetle, but seems less effective for lesion nematodes and diseases. Many of the studies are well-designed (randomized and replicated trials) and the evidence is of good quality so the certainty is relatively high. Some studies showed increases in pest species in certain rotations, so there are potential negative side-effects.

***Key things to note:***

The evidence base evaluated here excludes a number of studies on using crop rotation to address the pests including potato cyst nematodes and wire worms. The evidence is limited to America and Canada, so broader research is needed.

**Use pesticides only when pests or crop damage reach threshold levels**

**Description:** Switching from conventional, preventative pesticide applications (e.g. spraying every week, month or season) to a regime that monitors pest numbers or crop damage and applies pesticides only when these reach economically damaging levels (thresholds). Spraying regimes can be evaluated by assessing the number of occasions on which pests reached economically damaging levels after decisions to spray/not spray. Strategically timing insecticide sprays to coincide with periods of likely pest abundance or natural enemy susceptibility (anticipated in advance based on prior knowledge, experience or predictive models) is included in 'Alter the timing of insecticide use'. We use the term ‘pesticide’ to refer to insecticides (covering the majority of the evidence), fungicides and other chemicals to control non-plant organisms.

| **Practice** | **Effectiveness** | **Certainty of evidence** | **Negative side-effects** | **Category** |
| --- | --- | --- | --- | --- |
| [Use pesticides only when pests or crop damage reach threshold levels](http://www.conservationevidence.com/actions/750) | 39 | 30 | 20 | Unknown effectiveness |

**Crops studied** were barley, broccoli, cabbages, cauliflower, celery, cocoa, cotton, grape, peanut, potato, rice, tomato and wheat.

**Summary of discussion points:**

Effectiveness was scored low-middling by many because a lot of the benefits reported in the evidence synopsis related to reduced costs and pesticide inputs, rather than enhanced natural pest control. The evidence showed that using thresholds for pesticide application still offered effective chemical pest regulation but there was little evidence that this approach enhanced natural pest regulation; only one study directly measured the effect on natural enemy populations while the rest recorded impacts on pest numbers or damage, hence the certainty is fairly low.

***Key things to note:***

There is a significant body of evidence that shows that a) using pesticides reduces natural enemy populations, and b) that natural enemies deliver natural pest control, which would *suggest* that this practice should enhance natural pest control. However, these studies are not included within the evidence synopsis which includes only direct empirical study of the effect of the practice.

The type of pesticide used will affect the outcomes of this practice; broad spectrum compounds such as endosulfan and lindane will strongly reduce invertebrate numbers regardless of whether or not application is delayed.

**Incorporate parasitism rates when setting thresholds for insecticide use**

**Description:** Monitoring parasitism rates of pests by natural enemies and adjusting thresholds for insecticide use accordingly. Conventional threshold-based insecticide use (such as in integrated management) monitors pest populations or crop damage to schedule insecticide applications, but may not consider the action of natural enemies. Parasitoids can reduce pest populations, but there may be a lag between pest population increase and parasitoid population increase. If pests are killed by insecticides during this lag period, parasitoids may also be killed, preventing the parasitoid population from increasing and limiting the ecosystem service they provide. Monitoring parasitism rates to decide whether or not and when to spray is intended to avoid this and reduce unnecessary use of insecticides.

| **Practice** | **Effectiveness** | **Certainty of evidence** | **Negative side-effects** | **Category** |
| --- | --- | --- | --- | --- |
| [Incorporate parasitism rates when setting thresholds for insecticide use](http://www.conservationevidence.com/actions/726) | 50 | 10 | 5 | Unknown effectiveness |

**The crop studied** was tomato

**Summary of discussion points:**

The single study presented in the synopsis is well designed and shows a positive effect of the practice in enhancing natural pest regulation. The mechanism is clear and the results indicate an overall benefit, so there is a relatively high effectiveness score. No information is given on any side-effects.

***Key things to note:***

This assessment is based on a single study on tomatoes in New Zealand, so the certainty of evidence is very low and wider applicability of the practice is unknown. There is a need for significant further research for using the practice in other contexts. This practice is the basis of the ‘no spray for 40 days’ strategy that is commonly used by rice farmers in Asia. Spraying is delayed to allow pest-egg parasitoid populations to establish. Evidence from this context is not included in the evidence synopsis.

**Alter the timing of insecticide use**

**Description:** Applying insecticides at different dates in the growing season or at different times during the cropping cycle. Sprays can be reduced or avoided during periods of natural enemy vulnerability to reduce impacts on the ecosystem service they provide, although many studies test different dates simply to time spraying with periods of likely pest abundance or crop damage. Some of the evidence relates to chemicals now widely removed from use, and readers should bear in mind that using more selective insecticides may also allow greater flexibility in the timing of applications (‘Use more selective pesticides’ will be included in future synopses). Using historical information on pest population characteristics and crop susceptibility to time insecticides applications is included here. Informing spraying decisions by monitoring pests or crop damage within the present cropping season is included in 'Use pesticides only when pests or crop damage reach threshold levels'.

| **Practice** | **Effectiveness** | **Certainty of evidence** | **Negative side-effects** | **Category** |
| --- | --- | --- | --- | --- |
| [Alter the timing of insecticide use](http://www.conservationevidence.com/actions/723) | 40 | 28 | 13 | Unknown effectiveness |

**Crops studied** were aubergine, barley, maize, pear, stringbean.

**Summary of discussion points:**

There is some evidence that this practice can lead to enhanced natural enemy abundance and a subsequent reduction in pest numbers. The studies are well-designed and there is relatively good global coverage. However, the number of studies is quite small and many did not measure natural enemy numbers, leading to a low certainty score. Overall, the practice shows potential, but effects and appropriate timing are highly context specific, and there is a need for further research.

***Key things to note:***

Implementing this practice effectively will require context-specific planning based on the timing of pest colonisation, natural enemy life stages and incidence of damage, for a given situation.

The effectiveness will also depend on the type of insecticide used. Applying a narrow-spectrum compound with short-term environmental persistence could increase the effectiveness of the practice whilst reducing the potential for adverse side-effects. Some of the evidence in the synopsis relates to chemicals that are widely removed from use.

**Delay herbicide use**

**Description:** Delaying herbicide application dates within a growing season may improve natural pest control as this encourages weeds to grow early in the season, providing habitat and resources to help natural enemy populations develop. These weeds may also divert generalist pests (those with broad habitat or resource requirements) that would otherwise reach the crop.

| **Practice** | **Effectiveness** | **Certainty of evidence** | **Negative side-effects** | **Category** |
| --- | --- | --- | --- | --- |
| [Delay herbicide use](http://www.conservationevidence.com/actions/774) | 20 | 25 | 50 | Unknown effectiveness |

**Crops studied were beet and oilseed.**

**Summary of discussion points:**

There was some evidence that delaying herbicides to allow weeds to persist could enhance natural enemy numbers. However effects on pest numbers were very variable. The practice leads to an overall increase in the abundance of weeds, which are classed as a pest in this assessment. Additionally, there was an indication that weeds could support an increase in herbivorous insect species, which may cause crop damage. Therefore, overall effectiveness of this practice for enhancing natural pest regulation was judged to be low, and the potential negative side-effects were high. However, the certainty of evidence about how effective the practice is was low as results varied significantly between studies and sites.

***Key things to note:***

Weeds are included within the definition of pest. This practice has the potential to be effective in some context but the effects vary between crop types and sites. Coverage of evidence for this practice is very limited; there are only four studies in two crops.

**Use alley cropping**

**Description:** Agroforestry practice in which crops are grown between hedgerows or tree lines planted at regular intervals across crop fields or along slope contours. Hedges may be pruned and the foliage used as mulch or green manure on the adjacent crop alleys. The technique may control weeds and insect pests in a number of ways, for example by modifying the field’s climate, disrupting pest movement and weed growth, increasing crop vigour, providing habitat for natural enemies and using the insecticidal properties of hedgerow foliage. Studies that plant/allow trees around the edges of fields are included in 'Plant new hedges'.

| **Practice** | **Effectiveness** | **Certainty of evidence** | **Negative side-effects** | **Category** |
| --- | --- | --- | --- | --- |
| [Use alley cropping](http://www.conservationevidence.com/actions/718) | 15 | 35 | 50 | Unknown effectiveness |

**Crops studied were alfalfa, barley, cowpea, maize, pea, rice and wheat.**

**Summary of discussion points:**

Alley cropping can be highly effective in enhancing natural pest regulation when the alley crop species is chosen to suit the crop and pest type. However, effects on the abundance of natural enemies and pests were inconsistent among the studies in the synopsis. Overall, a good number of well-designed studies show no strong enhancement of natural pest regulation by this practice. This gave a low effectiveness score and a middling certainty score. Several of the studies showed that alley cropping could lead to an increase in some pest species, and there was some indication of crop damage and a potential reduction in yield and profit, leading to a high score for negative side-effects.

***Key things to note:***

The effectiveness of alley cropping is highly context dependent. It has the scope to be very effective when it is applied in a targeted way i.e. when the alley crop species is suited to the main crop species. There are also potential co-benefits and other reasons for implementing this practice, including erosion control and enhancing pollinator populations.

**Plant new hedges**

**Description:** Hedges or windbreaks are lines of trees or shrubs grown along the margins of crop and pasture fields or along orchard and plantation boundaries. Growing trees in rows within fields is included under 'Use alley cropping'. Ground-dwelling invertebrates (such as ground beetles and spiders) are frequently surveyed using pitfall traps – small pots buried in the ground up to their rim and left empty or filled with liquid preservatives or water. Pitfall trap measurements relate to both the abundance of beetles and their levels of activity on the ground, therefore studies refer to ‘activity densities’.

| **Practice** | **Effectiveness** | **Certainty of evidence** | **Negative side-effects** | **Category** |
| --- | --- | --- | --- | --- |
| [Plant new hedges](http://www.conservationevidence.com/actions/752) | 20 | 19 | 20 | Unknown effectiveness |

**Crops studied were barley, beans, maize and wheat.**

**Summary of discussion points:**

Some studies show that this practice enhances natural enemy numbers within the planted hedgerows, but very few measure natural enemy abundance actually within the crop. Therefore the certainty that this practice enhances natural pest regulation in the crop is limited. The evidence suggests that there is little impact beyond the close proximity of the hedge. For the practice to be effective the hedge species must be suited to the crop it is implemented in; for example, annual and perennial plants will support different pest and natural enemy species. The scale at which this is implemented is very important; in smaller fields, natural enemies may venture out from the hedgerow and reach a larger proportion of the crop than in larger fields. There is some evidence that hedges may also support pest species.

***Key things to note:***

This practice has potential to be effective if the hedge species, scale and layout are carefully suited to the context. There is a need for more research to measure effects on natural enemy numbers and pest regulation within the crop itself. The potential to use the practice in woody, perennial crops should be investigated.

**Allow natural regeneration of ground cover beneath perennial crops**

**Description:** This includes studies allowing the natural regeneration of weeds beneath perennial crops to enhance natural enemy populations. This includes studies testing the impact of tillage versus no tillage (or other types of soil disturbance) or herbicide versus no herbicide under perennial crops, where these practices are used to control weeds. Studies using naturally regenerated ground cover as a control treatment to compare with other practices (e.g. 'Grow plants that provide nectar or pollen resources' and 'Grow plants that provide supplementary prey for natural enemies') are not included here. Ground-dwelling invertebrates are frequently surveyed using pitfall traps – small pots buried in the ground up to their rim and left empty or filled with liquid preservatives or water.

| **Practice** | **Effectiveness** | **Certainty of evidence** | **Negative side-effects** | **Category** |
| --- | --- | --- | --- | --- |
| [Allow natural regeneration of ground cover beneath perennial crops](http://www.conservationevidence.com/actions/720) | 35 | 29 | 20 | Unknown effectiveness |

**Crops studied were apple, grape, lemon, olive and pear.**

**Summary of discussion points:**

There is some evidence that this practice can enhance the number and diversity of natural enemies, at least on the ground. However, this does not necessarily translate to enhanced numbers in the crop trees or vines. Species encouraged by ground vegetation may be unlikely to enter trees crops. The effect will depend on what vegetation species regenerate and the evidence suggests effects vary between natural enemy taxa groups and pest species. A good proportion of the studies presented are well-designed (randomised, replicated, controlled trials) but show variable results so certainty of evidence is low.

***Key things to note:***

This practice refers to natural regeneration both directly beneath the crop and in alley ways between crops.

**Isolate colonies of beneficial ants**

**Description:** This action involves pruning perennial crop trees to isolate ant colonies living in the tree canopy. Where ants act as natural predators, this action may improve pest control by reducing the time, energy and ant population losses incurred when rival ant colonies interact and viciously fight each other. This differs from the action 'Exclude ants that protect pests' for managing ants that limit rather than benefit natural pest control (for inclusion in a future synopsis).

| **Practice** | **Effectiveness** | **Certainty of evidence** | **Negative side-effects** | **Category** |
| --- | --- | --- | --- | --- |
| [Isolate colonies of beneficial ants](http://www.conservationevidence.com/actions/773) | 60 | 19 | 0 | Unknown effectiveness |

**The crop studied** was cashew.

**Summary of discussion points:**

The single study indicated that this practice was very effective and led to more natural enemies, fewer pests and increased yields. The study is well-designed and shows good evidence for the effect on several pest species. However, there is only one study so certainty is low. It is also a very specific practice and its wider applicability is uncertain.

***Key things to note:***

This assessment is only based on one study. The practice shows potential but the wider applicability is unclear and more research is needed.

**Delay mowing or first grazing date on pasture or grassland**

**Description:** This action involves delaying mowing or the onset of grazing on grasslands until later in the year. This may reduce damage to insect and spider natural enemy populations (or increase damage to pests) at sensitive points in their lifecycles, such as before overwintered individuals begin breeding. Ground-living invertebrates can be sampled by suction sampling, using a vacuum to suck-up and collect specimens for a given time or area of ground.

| **Practice** | **Effectiveness** | **Certainty of evidence** | **Negative side-effects** | **Category** |
| --- | --- | --- | --- | --- |
| [Delay mowing or first grazing date on pasture or grassland](http://www.conservationevidence.com/actions/131) | 5 | 20 | 15 | Unknown effectiveness |

**Crops studied** were barley, bird’s-foot trefoil, clovers, fescues, rapeseed, ryegrass, other grasses and wheat.

**Summary of discussion points:**

There is little consistent evidence that this practice will benefit natural enemies or enhance natural pest control. Some studies also indicate that it could reduce natural enemy numbers. There was also evidence that plant-feeding herbivores could increase (although it was not clear whether these were pests). The practice was judged to have low effectiveness in enhancing natural pest regulation. Much of the evidence was judged to be measuring general biodiversity rather than natural pest regulation. Results were also highly variable and no clear trend in effectiveness is obvious so certainty is low.

***Key things to note:***

**Create beetle banks**

**Description:** Beetle banks are raised strips which run through a field, typically planted with grasses. They primarily serve as an overwintering habitat for beetles, which provide pest control in the spring, but may also harbour other natural enemies. By dividing the field, beetle banks reduce the distance that predators have to travel to reach the centre of the crop, a potential problem if overwintering habitat occurs only at the field edge. Beetles are frequently surveyed using pitfall traps, but these measurements relate to both the abundance of beetles and their levels of activity on the ground; pitfall trap data therefore refer to ‘activity densities’.

| **Practice** | **Effectiveness** | **Certainty of evidence** | **Negative side-effects** | **Category** |
| --- | --- | --- | --- | --- |
| [Create beetle banks](http://www.conservationevidence.com/actions/729) | 25 | 60 | 10 | Unlikely to be beneficial |

**Crops studied** were barley, field bean, maize, oats, pasture, pea, radish, rapeseed, soybean and wheat.

**Summary of discussion points:**

The evidence shows that beetle banks can lead to an increase in natural enemies and a reduction in pests in, or close to, the banks. The studies in the synopsis tended not to measure the natural enemies within the crop. However, the few studies that did found that natural enemies only penetrated a short distance into the crop and only for a limited period of time. Enhancement of pest control within the crop is not strongly demonstrated in the evidence. The effectiveness is therefore scored relatively low. The synopsis includes a large number of well-designed studies showing relatively consistent results. However, there is a need for further research into the effect of beetle banks *within* the crop itself.

***Key things to note:***

The evidence in the synopsis shows this practice can increase natural enemy numbers within the banks, but there is a lack of research measuring the effects on natural pest regulation within the crops themselves. There may be evidence for this that has not been captured in the synopsis. It was suggested that beetle banks may be most beneficial as part of an Integrated Pest Management scheme, rather than used alone, as they are the studies included here.

**Incorporate plant remains into the soil that produce weed-controlling chemicals**

**Description:** Weeds can be suppressed by amending the soil with plant residues that produce allelopathic chemicals (biological chemicals that affect the growth of other organisms) as they decay. Plant residues are typically incorporated into soils by ploughing or rotavation. In some cases allelopathic plants may be grown as cover crops prior to being incorporated into the soil (green manuring). We consider this to be part of the ecosystem service where these plants can be grown in the ecosystem in question (for example on farms in that region). Applying plant residues to the soil surface is part of the action 'Add mulch to crops' and incorporating plant residues for inducing soil suppression of pests and pathogens is considered part of 'Amend the soil with fresh plant material or crop residues' (practices for inclusion in future synopses).

| **Practice** | **Effectiveness** | **Certainty of evidence** | **Negative side-effects** | **Category** |
| --- | --- | --- | --- | --- |
| [Incorporate plant remains into the soil that produce weed-controlling chemicals](http://www.conservationevidence.com/actions/728) | 39 | 47 | 30 | Likely to be ineffective or to have adverse side-effects |

**Crops studied** were beans, cotton, maize, rice and wheat.

**Summary of discussion points:**

The practice can be very effective in some contexts. Effects are weed and crop dependent and vary significantly between the systems studied in the evidence base. Some evidence showed an increase in pest species, and there was some indication of reduced yields so the potential for negative side-effects is relatively high. This practice can reduce pests directly, so it is uncertain whether observed pest reductions are due to this, or whether the practice actually enhances natural pest control. Otherwise, the evidence base comprises a reasonable number of well-designed studies, so the certainty of evidence is middling.

***Key things to note:***

Can be very effective in specific contexts. Depends on crop type, soil type, pest species etc.

**Use grazing instead of cutting for pasture or grassland management**

**Description:** Natural pest control in pastures can be affected by different methods of management and harvesting. Grazing may be less damaging to natural enemies and more suitable for some pest- or disease-resistant crop varieties than cutting. Direct effects of domestic livestock on pests (e.g. mortality by grazing and trampling) are not considered part of the natural ecosystem service of pest control but are summarized here if studies measured these effects while carrying out the practice. The intensity of grazing and frequency of cutting are often important factors and the practices ‘Reduce grazing intensity on grassland’ and ‘Reduce frequency of cutting on grassland or grass margins’ will be covered in future synopses. Ground-living invertebrates can be sampled by suction sampling, using a vacuum to suck-up and collect specimens for a given time or area of ground.

| **Practice** | **Effectiveness** | **Certainty of evidence** | **Negative side-effects** | **Category** |
| --- | --- | --- | --- | --- |
| [Use grazing instead of cutting for pasture or grassland management](http://www.conservationevidence.com/actions/885) | 10 | 45 | 40 | Likely to be ineffective or to have adverse side-effects |

**Crops studied** were alfalfa, cock’s-foot, perennial ryegrass, other grasses and white clover.

**Summary of discussion points:**

There was no clear benefit to natural enemy numbers or pest abundance from a large number of long-term, replicated, controlled studies. Most studies found no effects or mixed effects. Some studies showed a reduction in natural enemies and an increase in pests. Therefore the effectiveness of this practice in enhancing natural pest regulation was judged to be low with potential for negative side-effects.

***Key things to note:***

Evidence mainly from rye grass systems.

**Use mixed pasture**

**Description:** This involves growing more than one species of forage crop (grasses and legumes) in a pasture to control invertebrate or weed pests in pastoral farmland. The use of mixed pastures to suppress pests in arable crops is not included here but relevant to other practices, e.g. ‘Include plants that are repellent or suppressive to pests in crop rotations’ and ‘Grow cover crops that are repellent or suppressive to pests when the field is empty’ (for inclusion in a future synopsis).

| **Practice** | **Effectiveness** | **Certainty of evidence** | **Negative side-effects** | **Category** |
| --- | --- | --- | --- | --- |
| [Use mixed pasture](http://www.conservationevidence.com/actions/721) | 35 | 45 | 20 | Likely to be ineffective or to have adverse side-effects |

**Crops studied** were alfalfa, bird’s-foot trefoil, chicory, cicer milkvetch, clovers, fescues, oats, plantain, ryegrass, other grasses, other legumes, rapeseed and turnip.

**Summary of discussion points:**

There is some evidence to suggest using mixed pasture leads to lower weed density and diversity and fewer other pests. However, effects on natural enemies and pests are very mixed and system-specific. The evidence comes from several well-designed trials (replicated, long-term) so certainty is relatively high. A couple of studies indicate that the practice can lead to increased pests, so there are some potential negative side effects. There is some evidence that the practice can result in higher yields. However, this is likely to be because the mixes are designed for boosting yield, rather than because natural pest control has been enhanced and crop damage reduced.

***Key things to note:***

Evidence is available for several crop species, but all studies are from North America and Canada so effects of the practice elsewhere are uncertain.

Using mixed pasture is generally designed to enhance yield. If the mixes were specifically designed to enhance natural pest control, this practice could be more effective in delivering this service.


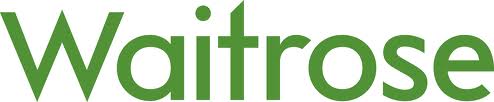
This assessment process was funded by Waitrose plc.

# Appendix 1: Background to study and methodology

Natural pest regulation is when a ‘natural enemy’ species suppresses one or more species that cause crop damage or yield loss. It can be a valuable service in agriculture that can reduce the need for agrichemical inputs leading to potential advantages such as reduced costs, reduced reliance on restricted compounds and improved environmental conditions on farms. However, implementing an action that might not work is risky and identifying effective ways of boosting natural pest regulation can be challenging.

There is a large amount of scientific research providing evidence for the effectiveness of different practices for enhancing natural pest regulation. To make this information accessible to growers and farm advisors, the Conservation Science Group has synthesised over 175 studies into short summaries for each practice. These are available in the [Conservation Evidence Natural Pest Control Synopsis](http://www.conservationevidence.com/synopsis/download/10http:/www.conservationevidence.com/synopsis/download/10) and on the [Conservation Evidence](http://www.conservationevidence.com) website (also see hyperlinks throughout the document).

In this guide, we synthesise this information further to provide a quick reference guide that signposts the effectiveness of different practices for enhancing natural pest regulation, based on the information in the summarised scientific evidence. This was done by a process of expert assessment.

**Expert assessment**

We used a method based on the Delphi technique (described by Hutchings and Raine, 2006), in which a group of 16 experts on pest regulation completed multiple scoring and discussion exercises. Group members were chosen to represent a range of viewpoints and interests in agricultural pest regulation:

| **Expert group members** | |
| --- | --- |
| Dr Toby Bruce | Rothamsted Research |
| Phillip Effingham | Greentech Consultants |
| Melvyn Fidgett | Syngenta |
| Dr Michelle Fountain | East Malling Research |
| Dr Michael Garratt | University of Reading |
| Anthony Goggin | Linking Environment and Farming (LEAF) |
| Tony Harding | Worldwide Fruit |
| Dr Finbarr Horgan | International Rice Research Institute |
| Dr Mattias Jonsson | Swedish University of Agricultural Sciences |
| Vicky Kindemba | Buglife |
| Dr Steve Sait | University of Leeds |
| Dr Barbara Smith | Game and Wildlife Conservation Trust |
| Dr Eve Veromann | Estonian University of Life Sciences |
| Professor Felix Wäckers | BioBest, Belgium/University of Lancaster |
| Dr Andrew Wilby | University of Lancaster |
| Dr Stephanie Williamson | Pesticides Action Network UK |

The assessment was of summarised scientific evidence in the Conservation Evidence Natural Pest Control Synopsis. Based on this evidence, the group assessed:

1. The effectiveness of each practice in enhancing natural pest control
2. The strength of any potential negative side-effects associated with the practice
3. The certainty of the evidence about each practice in the synopsis
4. The coverage of the evidence in the synopsis

Group members first completed a survey in which they scored each of the practices for the 4 criteria above, based on the information in the synopsis (see detail of questions and scoring in Appendix 3). They then attended a workshop where they discussed the practices and the evidence in detail with the rest of the expert group. Based on any new perspectives and information they heard from other participants, each group member then entered final assessments for each of the practices. Important discussion points were recorded by a scribe. A median of the final scores was used to give an overall assessment of the practices, based on the evidence provided.

The scores have been used to put the practices into 6 indicative categories, based on categories used to classify clinical practices in medicine (see *Clinical Evidence Handbook,* BMJ 2013):

1. Beneficial (in enhancing natural pest regulation)
2. Likely to be beneficial
3. Trade-offs between benefits and adverse side-effects
4. Unknown effectiveness
5. Unlikely to be beneficial
6. Likely to be ineffective or to have adverse side-effects.

These offer a quick indication of how effective a practice might be in enhancing natural pest regulation, according to the evidence in the synopsis. The score thresholds used to categorise the interventions are shown below:

| **Categories** | **Score thresholds for categorisation** | | | **Notes** |
| --- | --- | --- | --- | --- |
|  | **Effectiveness** | **Certainty** | **Side-effects** |  |
| Beneficial | > 60 | >60 | <20 |  |
| Likely to be beneficial: criteria 1 | >60 | 40-60 | <20 | High effectiveness, lower certainty |
| Likely to be beneficial: criteria 2 | 40-60 | 40+ | <20 | Lower effectiveness, but good certainty |
| Trade-offs between benefits and adverse side-effects | ≥40 | ≥40 | 20+ |  |
| Unknown effectiveness |  | <40 |  |  |
| Unlikely to be beneficial | <40 | 40-60 | <20 |  |
| Likely to be ineffective or to have adverse side-effects: criteria 1 | <40 | >60 |  | Low effectiveness, high certainty (regardless of negative side-effects) |
| Likely to be ineffective or to have adverse side-effects: criteria 2 | <40 | 40+ | 20+ | Low effectiveness, negative side-effects (lower certainty about effectiveness) |

**Scope**

The 21 practices included here are a subset of a complete list of 92 practices for enhancing natural pest regulation on farmland (Appendix 2). Many in the subset were selected through consultation with groups of stakeholders from the food production industry, agricultural policy and academia (detail in Appendix 2). The remainder were chosen to represent the range of farming systems and different types of practice in the complete list. Some widely-used techniques, such as resistant crop varieties or flower margins, are not included in the current assessment, but are in the complete list. So far, time and resources have meant that we have been able to summarise evidence and conduct expert assessment for this subset of 21 practices. These include some techniques that may be considered ‘niche’, but which are valuable to consider as potential alternative ways to enhance natural pest regulation. We hope to extend the work to the remaining practices in the future.

**Interpreting this guide**

The expert assessment was of the evidence within the [Natural Pest Control Synopsis](http://www.conservationevidence.com/synopsis/download/10http:/www.conservationevidence.com/synopsis/download/10). The scores and categorisations in this document are made according to the information in these studies.

Each practice is described in a short definition at the start of its section. Some practices are similar to others in the assessment, or others included in the complete list of 92 practices to enhance natural pest control (Appendix 2). Please read the definitions carefully to be sure what the assessment refers to.

The focus here is on the effectiveness of the practices in *enhancing the delivery of natural pest regulation*. Many of the practices have potential co-benefits or are designed to deliver a management objective other than this (eg farmland biodiversity, soil fertility). However, these are not considered in the current assessment, and neither is the direct pest control effect of the practices.

The evidence assessed here relates to each of the practices implemented in isolation. In reality, it is likely that several would be used together in Integrated Pest Management schemes, and their combined effects may be different. Additionally, for several of the techniques, the evidence suggests that they may be effective in some contexts but not others. The overall category therefore may be ‘unknown effectiveness’ but for some particular crop systems, the practice may be beneficial. This will be noted under ‘key things to note’. Please refer to the [Natural Pest Control Synopsis](http://www.conservationevidence.com/synopsis/download/10http:/www.conservationevidence.com/synopsis/download/10) or use the hyperlinks within each section to find further information about each practice.

# Appendix 2: Complete list of 92 natural pest control practices

| **REDUCING AGRICULTURAL POLLUTION** | | **Num. of studies** |
| --- | --- | --- |
| ***Pesticides and herbicides*** | |  |
| 1 | Reduce pesticide use * | 404 |
| 2 | Use more selective pesticides | 225 |
| 3 | Provide refuges from spraying for natural enemies | 2 |
| 4 | Leave headlands in fields unsprayed (conservation headlands) | 8 |
| 5 | Use chemical application techniques that reduce the impact on natural enemies | 30 |
| 6 | Use pesticides only when pests or crop damage reach threshold levels *^ | 29 |
| 7 | Incorporate parasitism rates when setting thresholds for insecticide use ^ | 1 |
| 8 | Alter the timing of insecticide use *^ | 13 |
| 9 | Reduce herbicide use * | 108 |
| 10 | Delay herbicide use ^ | 4 |
| 11 | Avoid using genetically modified insecticidal or herbicide-resistant crops | 48 |
|  |  |  |
| ***Fertilizers*** | |  |
| 12 | Reduce mineral fertilizer use * | 266 |
| 13 | Use organic rather than mineral fertilizers | 90 |
|  |  |  |
| ***Reducing chemicals in general*** | |  |
| 14 | Reduce pesticide, herbicide or fertilizer use generally (including integrated management methods) * | 182 |
| 15 | Convert to organic farming * | 82 |
|  |  |  |
| **ALL FARMING SYSTEMS** | |  |
| ***Manage habitat and food*** | |  |
| 16 | Grow plants that provide nectar or pollen resources | 128 |
| 17 | Grow plants that provide supplementary prey for natural enemies | 22 |
| 18 | Grow plants that provide shelter, habitat or other resources for natural  enemies * | 106 |
| 19 | Provide grass buffer strips/margins around arable or pasture fields * | 31 |
| 20 | Provide refuges for natural enemies | 8 |
| 21 | Use alley cropping ^ | 10 |
| 22 | Plant new hedges ^ | 6 |
| 23 | Include short rotation coppice in the agricultural landscape | 2 |
| 24 | Provide supplementary food for natural enemies | 7 |
| 25 | Use mass-emergence devices to increase natural enemy populations ^ | 1 |
|  |  |  |
| ***Manage crops*** | |  |
| 26 | Increase whole-farm crop diversity | 4 |
| 27 | Plant more than one crop per field * | 570 |
| 28 | Change the density at which crops are planted | 171 |
| 29 | Use grafting to combine different crop varieties | 6 |
| 30 | Use crop varieties with different timings or rates of growth | 29 |
| 31 | Use crop varieties that resist or suppress pests, diseases or weeds * | 383 |
| 32 | Induce plant defences against pests and pathogens | 59 |
| 33 | Apply organic liquids (e.g. crop and compost extracts) to crop foliage | 33 |
| 34 | Add mulch to crops * | 216 |
| 35 | Reduce tillage * | 375 |
| 36 | Reduce mechanical weed control | 74 |
| 37 | Leave part of the crop or pasture unharvested or uncut ^ | 12 |
| 38 | Reduce frequency of cutting on pasture, grassland or grass margins | 23 |
| 39 | Alter irrigation regime | 129 |
|  |  |  |
| ***Control insect distribution*** | |  |
| 40 | Plant and manage trap crops to attract pests away from crop | 176 |
| 41 | Use crop types and varieties that attract natural enemies or enhance their effectiveness | 21 |
| 42 | Grow non-crop plants that produce chemicals that attract natural enemies *^ | 6 |
| 43 | Use chemicals to attract natural enemies ^ | 15 |
|  |  |  |
| **ARABLE FARMING** | |  |
| ***Manage habitat*** | |  |
| 44 | Create uncropped field margins or plots by allowing natural regeneration * | 25 |
| 45 | Create beetle banks ^ | 18 |
| 46 | Provide bird perches in fields | 4 |
|  |  |  |
| ***Manage crops*** | |  |
| 47 | Intercrop with plants that are repellent or suppressive to pests or weeds * | 128 |
| 48 | Grow one crop using a mixture of varieties within a field | 6 |
| 49 | Use crop rotation *^ ^1^ | 252 |
| 50 | Include plants that are repellent or suppressive to pests in crop rotations | 62 |
| 51 | Incorporate fallow periods into crop rotation | 75 |
| 52 | Incorporate leys into crop rotation | 24 |
| 53 | Use relay intercropping | 8 |
| 54 | Grow cover crops when the field is empty | 83 |
| 55 | Grow cover crops that are repellent or suppressive to pests when the field is empty | 22 |
| 56 | Grow crops in strips within a cover crop | 3 |
| 57 | Grow cover crops beneath the main crop (living mulches) or between crop  rows * | 103 |
| 58 | Leave overwinter stubbles | 1 |
| 59 | Reduce burning of crop remains | 15 |
| 60 | Alter timing of sowing or harvesting | 445 |
|  |  |  |
| ***Control insect distribution*** | |  |
| 61 | Combine trap and repellent crops in a push-pull system ^ | 13 |
|  |  |  |
| ***Soil mulch and amendments*** | |  |
| 62 | Mulch with plants that produce pesticidal fumes as they decay (such as mustard) | 9 |
| 63 | Incorporate pesticidal plant material into the soil | 70 |
| 64 | Incorporate plant remains into the soil that produce weed-controlling chemicals ^ | 10 |
| 65 | Amend the soil with fresh plant material or crop residues | 62 |
| 66 | Amend the soil with crops grown as green manures | 99 |
| 67 | Amend the soil with processed plant materials | 132 |
| 68 | Amend the soil with manures and agricultural composts | 227 |
| 69 | Amend the soil with organic processing wastes or their composts | 93 |
| 70 | Amend the soil with municipal wastes or their composts | 44 |
| 71 | Amend the soil with composts not otherwise specified | 126 |
| 72 | Amend the soil with non-chemical minerals and mineral wastes | 16 |
| 73 | Amend the soil with formulated chemical compounds | 39 |
| 74 | Amend the soil with materials not otherwise specified | 45 |
|  |  |  |
| **PERENNIAL FARMING** | |  |
| ***Manage crops and ground cover*** | |  |
| 75 | Allow natural regeneration of ground cover beneath perennial crops ^ | 13 |
| 76 | Grow cover crops under perennial tree crops | 50 |
| 77 | Cut cover crops and place in perennial tree crops to move natural enemies into the canopy | 2 |
| 78 | Grow pest-suppressive crops prior to planting perennial crops | 1 |
|  |  |  |
| ***Manage ants*** | |  |
| 79 | Exclude ants that protect pests ^ | 8 |
| 80 | Isolate colonies of beneficial ants ^ | 1 |
|  |  |  |
| **LIVESTOCK FARMING AND PASTURE** | |  |
| ***Livestock breeds*** | |  |
| 81 | Use resistant livestock breeds | 1 |
|  |  |  |
| ***Manage pastures*** | |  |
| 82 | Restore or create low-input grassland | 0 |
| 83 | Reduce management intensity on pasture or permanent grassland | 4 |
| 84 | Reduce grazing intensity on pasture or grassland | 44 |
| 85 | Delay mowing or first grazing date on pasture or grassland ^ | 11 |
| 86 | Raise mowing height on pasture or grassland | 4 |
| 87 | Use grazing instead of cutting for pasture or grassland management ^ | 8 |
| 88 | Cut noxious weeds to increase disease incidence | 2 |
| 89 | Grow plants that compete with damaging weeds ^ | 13 |
| 90 | Use mixed pasture ^ | 10 |
|  |  |  |
| ***Modify housing conditions*** | |  |
| 91 | Modify flooring in poultry houses to benefit natural enemies | 3 |
|  |  |  |
| ***Manage disease hosts*** | |  |
| 92 | Cull wildlife hosts of livestock disease | 7 |

* Featured in the top 10 practices as chosen by groups of experts in a replicated workshop exercise to prioritize the complete list. These practices were chosen by at least one of four groups.
^ Summarized in this synopsis.
^1^ Adapted to ‘Use crop rotation in potato farming systems’ for this synopsis.

***Identifying 21 practices for inclusion in the natural pest control synopsis***

The complete list of 92 practices to enhance natural pest control was developed from a list suggested by ecosystem service experts. These practices were refined and added to as we reviewed the literature on enhancing natural pest control. An international advisory board of seven experts (from academia, private-sector research and independent and charitable organisations) also commented on and added to the list.

Practices were included if they were practices that farmers or land-managers would realistically be prepared to or could do. We included practices regardless of whether they had already been adopted or whether or not evidence for their effectiveness already existed.

The great volume of relevant studies and the short timescale of the Knowledge Exchange Programme precluded us from summarizing all of the literature, therefore this synopsis presents the evidence from 157 studies covering 21 selected practices. To prioritize the practices we asked stakeholders from the food production industry, agricultural policy and academia to select their top 10 practices. A prioritisation exercise (using a modification of the Delphi process) was repeated four times with different groups of eight experts, during a workshop in Paris (in collaboration with FRB) in mid-January 2013. Participants came from several western European countries and were asked to vote on their personal top 10 practices and then agree the group’s final top 10 by consensus. The priorities identified were encouragingly consistent between the four groups and are marked in the Appendix 1 Table above.

Four priority practices (each with fewer than 100 studies) are included in the synopsis, balancing the expert’s priorities with this project’s time constraints. The 17 other practices were chosen to represent all farming systems and the variety of different types of practice in the complete list of practices. ‘Use crop rotation’ was a priority action with a very large literature and therefore for the purpose of this synopsis we consider the evidence for potato farming systems only.

# Appendix 3: Explanation of scoring and detail of experts’ scores for each practice

For each of the practices, members of the expert group were asked to read the relevant section of the Conservation Evidence Natural Pest Control Synopsis and answer the following questions using the given scoring scales:

1. **Based on the evidence in the natural pest control synopsis, how effective is this intervention in enhancing natural pest regulation in agricultural systems?**
   Score between 0 and 100 where: 0 = Ineffective, 25 = moderately effective, 50 = effective, 75 = very effective, 100 = always effective
2. **Based on the evidence in the synopsis, are there any potential negative side-effects of this intervention?** Score between 0 and 100 where: 0 = none, 25 = minor, 50 = some, 75 = large, 100 = major
3. **What is the certainty of evidence for the effectiveness of this intervention in enhancing natural pest regulation in the systems covered in the synopsis?** Score between 0 and 100 where: 0 = no certainty; no useful evidence available, 25 = low certainty; little quality evidence, 50 = moderate certainty; some quality evidence, 75 = high certainty; a lot of quality evidence, 100 = complete certainty; high quality evidence.
4. **How complete is the coverage of the evidence within the synopsis? (i.e. how complete is the coverage of countries, crop and farm systems relative to all of the contexts in which the intervention *could* be used).** Score between 0 and 100 where: 0 = no coverage, 25 = poor coverage, 50 = moderate coverage, 75 = Good coverage, 100 = complete coverage

| **Median group scores** | | | | | |
| --- | --- | --- | --- | --- | --- |
| **Practice** |  | **Final median score from expert group** | | | |
|  | **Section in synopsis** | **Effectiveness** | **Negative side-effects** | **Certainty of evidence** | **Coverage of evidence** |
| Combine trap and repellent crops in a push-pull system | 4.3 | 70 | 5 | 68 | 19 |
| Grow non-crop plants that produce chemicals that attract natural enemies | 3.4 | 68 | 0 | 40 | 36 |
| Use chemicals to attract natural enemies | 3.5 | 40 | 15 | 50 | 65 |
| Exclude ants that protect pests | 5.2 | 40 | 12 | 50 | 60 |
| Grow plants that compete with damaging weeds | 6.3 | 70 | 5 | 60 | 40 |
| Leave part of the crop or pasture unharvested or uncut | 3.3 | 45 | 25 | 50 | 15 |
| Use crop rotation in potato farming systems | 4.2 | 50 | 25 | 50 | 29 |
| Use pesticides only when pests or crop damage reach threshold levels | 2.1 | 39 | 20 | 30 | 40 |
| Delay mowing or first grazing date on pasture or grassland | 6.1 | 5 | 15 | 20 | 30 |
| Incorporate parasitism rates when setting thresholds for insecticide use | 2.2 | 50 | 5 | 10 | 5 |
| Alter the timing of insecticide use | 2.3 | 40 | 13 | 28 | 30 |
| Delay herbicide use | 2.4 | 20 | 50 | 25 | 20 |
| Use alley cropping | 3.1 | 15 | 50 | 35 | 40 |
| Plant new hedges | 3.2 | 20 | 20 | 19 | 19 |
| Use mass-emergence devices to increase natural enemy populations | 3.6 | 16 | 0 | 10 | 4 |
| Allow natural regeneration of ground cover beneath perennial crops | 5.1 | 35 | 20 | 29 | 40 |
| Isolate colonies of beneficial ants | 5.3 | 60 | 0 | 19 | 5 |
| Create beetle banks | 4.1 | 25 | 10 | 60 | 40 |
| Incorporate plant remains into the soil that produce weed-controlling chemicals | 4.4 | 39 | 30 | 47 | 40 |
| Use grazing instead of cutting for pasture or grassland management | 6.2 | 10 | 40 | 45 | 50 |
| Use mixed pasture | 6.4 | 35 | 20 | 45 | 30 |

# Appendix 4: Excluded practices

Two practices that appear in the full Natural Pest Control synopsis were excluded from the current assessment.

[**Convert to organic farming**](http://www.conservationevidence.com/actions/717)

This management option is included in the original Natural Pest Control Synopsis. However, due to time and resource constraints, and the very large number of studies found, only a subset of studies could be summarised in the synopsis. These are limited to direct experimental tests of converting to organic farming. Site comparison studies that compare organic with conventional farms are excluded from the synopsis. An Advisory Panel for the publication cautioned that this subsection of the full body of available evidence is not a reliable representation of the effects of converting to organic farming and therefore it was decided to exclude this practice from the current assessment.

[**Use mass-emergence devices to increase natural enemy populations**](http://www.conservationevidence.com/actions/775)
This practice relates to relocating natural enemies (or plant matter hosting natural enemies) from elsewhere in the same system. It ***does not*** cover the introduction of external natural enemies to the system i.e. biocontrol. During workshop discussions, the group voted to exclude this practice from further assessment because it was judged that the evidence captured in the synopsis was incomplete and not representative of the extent of information available for this practice. There is only a single study presented in the synopsis and it is from a non-farming context.
